# System-wide molecular dynamics of endothelial dysfunction in Gram-negative sepsis

**DOI:** 10.1186/s12915-020-00914-0

**Published:** 2020-11-24

**Authors:** Xavier Gallart-Palau, Aida Serra, Siu Kwan Sze

**Affiliations:** 1 School of Biological Sciences, Nanyang Technological University, 60 Nanyang Drive, Singapore, 637551 Singapore; 2 University Hospital Institut Pere Mata, Reus, Tarragona Spain; 3 Institut Investigació Sanitària Pere Virgili (IISPV), Reus, Tarragona Spain; 4 Centro de investigación Biomédica en Salud Mental CIBERSAM, Instituto de Salud Carlos III, Madrid, Spain; 5 IMDEA Food & Health Sciences Research Institute, +Pec Proteomics, Campus of International Excellence UAM+CSIC, Old Cantoblanco Hospital, 8 Crta. Canto Blanco, 28049 Madrid, Spain; 6 Proteored - Instituto de Salud Carlos III (ISCIII), Madrid, Spain

**Keywords:** Vascular beds, Lipopolysaccharide, Endothelial dysfunction, Inflammation, Infection, DISDIVO

## Abstract

**Background:**

Inflammation affecting whole organism vascular networks plays a central role in the progression and establishment of several human diseases, including Gram-negative sepsis. Although the molecular mechanisms that control inflammation of specific vascular beds have been partially defined, knowledge lacks on the impact of these on the molecular dynamics of whole organism vascular beds. In this study, we have generated an in vivo model by coupling administration of lipopolysaccharide with stable isotope labeling in mammals to mimic vascular beds inflammation in Gram-negative sepsis and to evaluate its effects on the proteome molecular dynamics. Proteome molecular dynamics of individual vascular layers (glycocalyx (GC), endothelial cells (EC), and smooth muscle cells (SMC)) were then evaluated by coupling differential systemic decellularization in vivo with unbiased systems biology proteomics.

**Results:**

Our data confirmed the presence of sepsis-induced disruption of the glycocalyx, and we show for the first time the downregulation of essential molecular maintenance processes in endothelial cells affecting this apical vascular coating. Similarly, a novel catabolic phenotype was identified in the newly synthesized EC proteomes that involved the impairment of protein synthesis, which affected multiple cellular mechanisms, including oxidative stress, the immune system, and exacerbated EC-specific protein turnover. In addition, several endogenous molecular protective mechanisms involving the synthesis of novel antithrombotic and anti-inflammatory proteins were also identified as active in EC. The molecular dynamics of smooth muscle cells in whole organism vascular beds revealed similar patterns of impairment as those identified in EC, although this was observed to a lesser extent. Furthermore, the dynamics of protein posttranslational modifications showed disease-specific phosphorylation sites in the EC proteomes.

**Conclusions:**

Together, the novel findings reported here provide a broader picture of the molecular dynamics that take place in whole organism vascular beds in Gram-negative sepsis inflammation. Similarly, the obtained data can pave the way for future therapeutic strategies aimed at intervening in specific protein synthesis mechanisms of the vascular unit during acute inflammatory processes.

## Background

Homeostasis in all systems of the human body depends, to a large extent, on the molecular and structural integrity of the cardiovascular system (CVS). This intricate and supportive system provides adaptive metabolic supplementation of nutrients, molecular messengers, and oxygen to cells, while it eliminates unwanted residues and sustains immunity [1, 2]. The CVS is formed by a vast network of vessels that vary in length, diameter, and function, with endothelial cell (EC) and glycocalyx (GC) layers at the inner areas of vasculature beds [2]. Additionally, arteries and veins are formed by vascular smooth muscle cells (SMC), a population of innervated cells with the ability to regulate vascular tone in conjunction with EC [3, 4].

Dysfunction of the endothelium is associated with the appearance and progression of the most severe human diseases, including sepsis, diabetes, stroke, dementia, and cancer [5–10]. Although these diseases are characterized by specific alterations of the endothelium, some of which have yet to be fully elucidated, inflammation has been defined as a core pathological mechanism affecting vascular beds in all these pathologies [11, 12]. Similarly, inflammation has been found to be a core mechanism of microvasculature disruption preceding organ dysfunction in sepsis [13]. Of note, recent epidemiological compilations indicate that the burden of sepsis exceeds that of cancer globally, and it has become the second-ranked global cause of death behind only cardiovascular diseases [14, 15].

Lipopolysaccharide (LPS), also known as endotoxin, is a bacterial molecule centrally implicated in the pathogenesis of severe sepsis and septic shock [16]. This circulating toxin has the ability to indicate the occurrence of sepsis in blood while activating the systemic release of a myriad of pro-inflammatory molecules [17]. These pro-inflammatory factors are known to disrupt vascular beds by promoting the apoptosis of EC, which in turn leads to edema formation and organ failure [18, 19]. Although LPS-induced disruption of the endothelial barrier has been thoroughly described in specific vascular beds, such as the lungs and liver, the comprehension of the molecular events that precede vascular bed disruption in severe sepsis remains poor. Similarly, further light needs to be shed on the effects of LPS throughout the whole organismal network of capillary beds. According to the recent literature review performed by Libert et al. [20], LPS has been used in one third of the most relevant studies that involve the use of animal models in sepsis. However, it has been admitted that sepsis is caused by a highly complex pathophysiology that cannot be fully mimicked using LPS in rodents [20]. The use of LPS, thus, should be limited to mimicking specific relevant clinical features of sepsis in a robust, quick, precise, and highly replicable manner, especially the severe inflammatory response that affects the endothelium in this disease and causes the appearance of fever, leukocytosis, and cytokine release, among other features [20].

Alterations in protein synthesis can be considered among the earliest molecular events of disease progression [21, 22]. Bacterial pathogenesis has recently been associated with alterations in protein synthesis in platelets and gut epithelial cells [23, 24], although little is still known about how the cell renewal mechanism becomes impaired in the whole organism vascular bed layers. Novel systems biology methods coupled to the study of whole organism vascular beds hold promise for advancing the understanding of the effects of sepsis on protein synthesis in vascular and capillary beds. Thus, in this study, we combined for the first time stable isotope labeling of mammals (SILAM) [25, 26] with differential systemic decellularization in vivo (DISDIVO) [27] to characterize the molecular dynamics of whole organism vascular beds in Gram-negative sepsis. SILAM, as initially reported by Yates, J.R. III, and colleagues [28] is based on the depletion of light proteins (proteins without isotope-labeled Lys) in the dietary protein source of the animal by substitution of these with heavy proteins (proteins with isotope-labeled Lys). Thus, all newly synthesized proteomes in the animal incorporate isotope-labeled Lys, which can in turn be easily identified/quantified by mass spectrometry [28]. Similarly, DISDIVO allows systemic decellularization of independent vascular layers and analysis of the vascular layers proteomes by systems biology [27]. Although optimization of the DISDIVO method demonstrated that the conditions used for the obtention of each independent vascular layer are the most experimentally appropriate, the possibility that cross-contamination between vascular mantles occurs in DISDIVO cannot be discarded. However, based on the extensive imaging and bioinformatics analysis performed on data generated by this method, the systematic approach of DISDIVO involving the entire vascular system seems able to compensate for these potential peculiarities. Our novel findings indicate that dramatic inhibition of protein synthesis and partial protein synthesis shift occur in whole organism vascular bed layers together with abnormal protein turnover in EC. Thus, the findings uncovered here highlight specific interference by endotoxemia in the regular molecular dynamics of the whole organism endothelium during acute inflammatory processes.

## Results

### Use of SILAM-DISDIVO for the study of whole organism vascular beds in Gram-negative sepsis

**Fig. 1:**
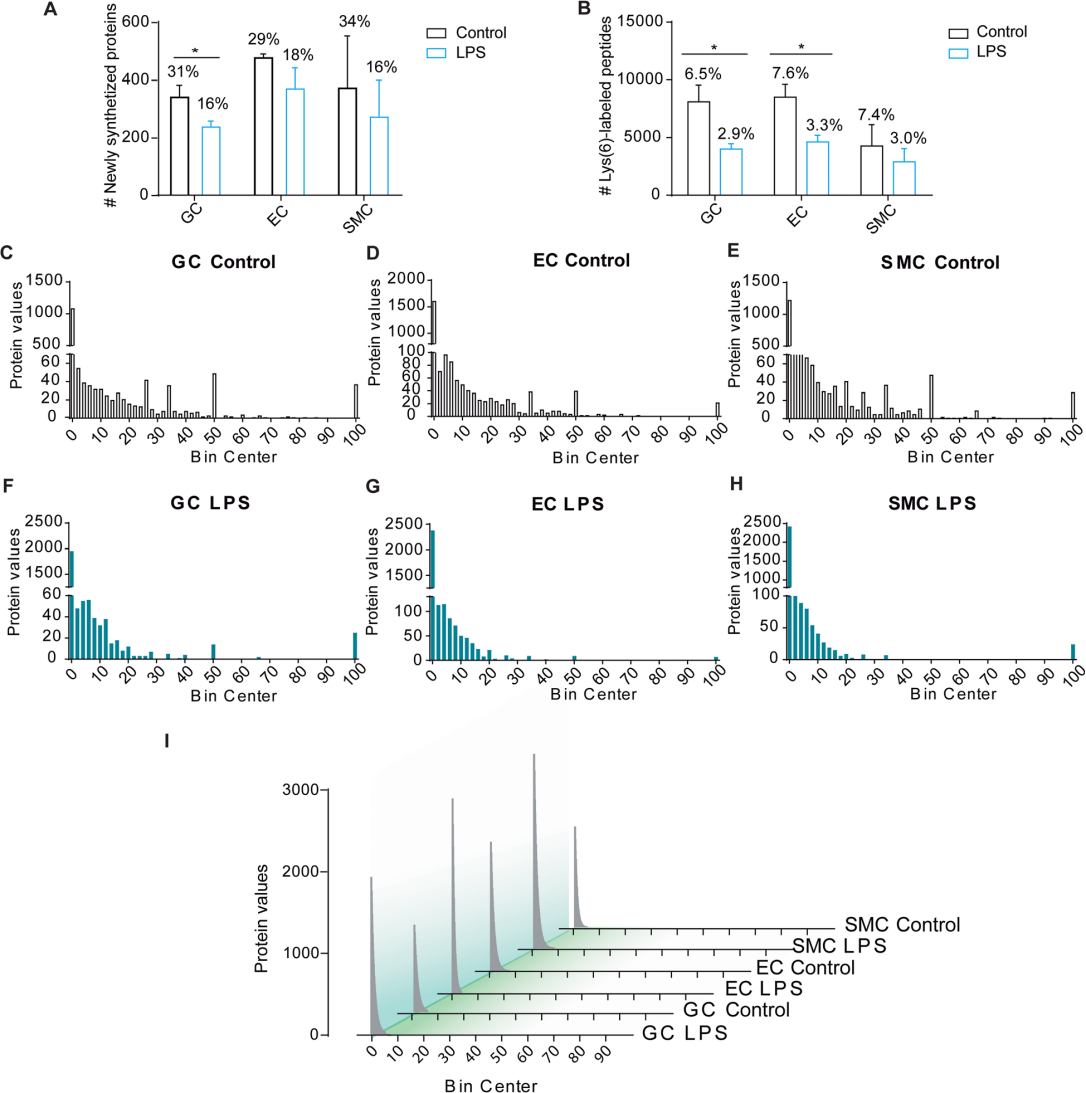
Validation of the SILAM model for the study of protein dynamics in severe inflammatory response. **a** Comparison of the number of SILAM-labeled proteins detected in glycocalyx (GC), endothelial cells (EC) and smooth muscle cells (SMC) proteomes after endotoxemia (LPS) versus Control. **b** Number of tryptic digested SILAM-labeled peptides detected in the three analyzed vascular beds (GC, EC, and SMC). **c**–**h** Frequency distribution curves for the incorporation of Lys(6) in individual proteins calculated for every vascular bed (GC, EC, and SMC) after LPS versus Control. **i** Adjusted curve comparison for the SILAM-labeled proteins in different vascular beds from Control and LPS-treated mice

To study the effect(s) of the endotoxin LPS on the proteome dynamics of whole organism vascular beds during sepsis, we made use of a SILAM model generated by replacing dietary Lys with the stable isotope Lys(6). Validation of DISDIVO fractions in whole SILAM mouse vascular beds (GC, EC, and SMC) revealed the proper incorporation of Lys(6) into newly synthesized proteins, as shown in Fig. 1. Lys(6) was incorporated on average in a total of 350 ± 112 newly synthesized proteins in vascular bed proteomes, which represented 31% of the total proteome in the GC in control mice, 29% of the total proteome in EC, and 34% of the total proteome in SMC in control mice. In addition, in Gram-negative sepsis, newly synthesized proteins represented only 16% of the total proteome in the GC, 18% of the total proteome in EC, and 16% of the total proteome in SMC (Fig. 1a, Additional File 1: Dataset 1 and Additional File 2: Dataset 2). Finally, the validated SILAM model also revealed the efficient averaged labeling of Lys(6) in a total of 5.1 ± 2.3% of all analyzed peptidomes (Fig. 1b).

### Molecular dynamics of whole organism vascular beds during Gram-negative sepsis

Detailed analysis of the molecular dynamics occurring in whole organism vascular beds during Gram-negative sepsis indicated that LPS challenge triggers a significant, rapid, and severe reduction in the molecular maintenance of the GC. Thus, a significant decrease in the total number of Lys(6)-labeled proteins detected in this vascular coating layer was observed, as shown in Fig. 1a. Similarly, a slight reduction in the total number of newly synthesized proteins was observed in the EC and SMC vascular layers in Gram-negative sepsis, although these differences did not reach statistical significance (Fig. 1a).

**Fig. 2:**
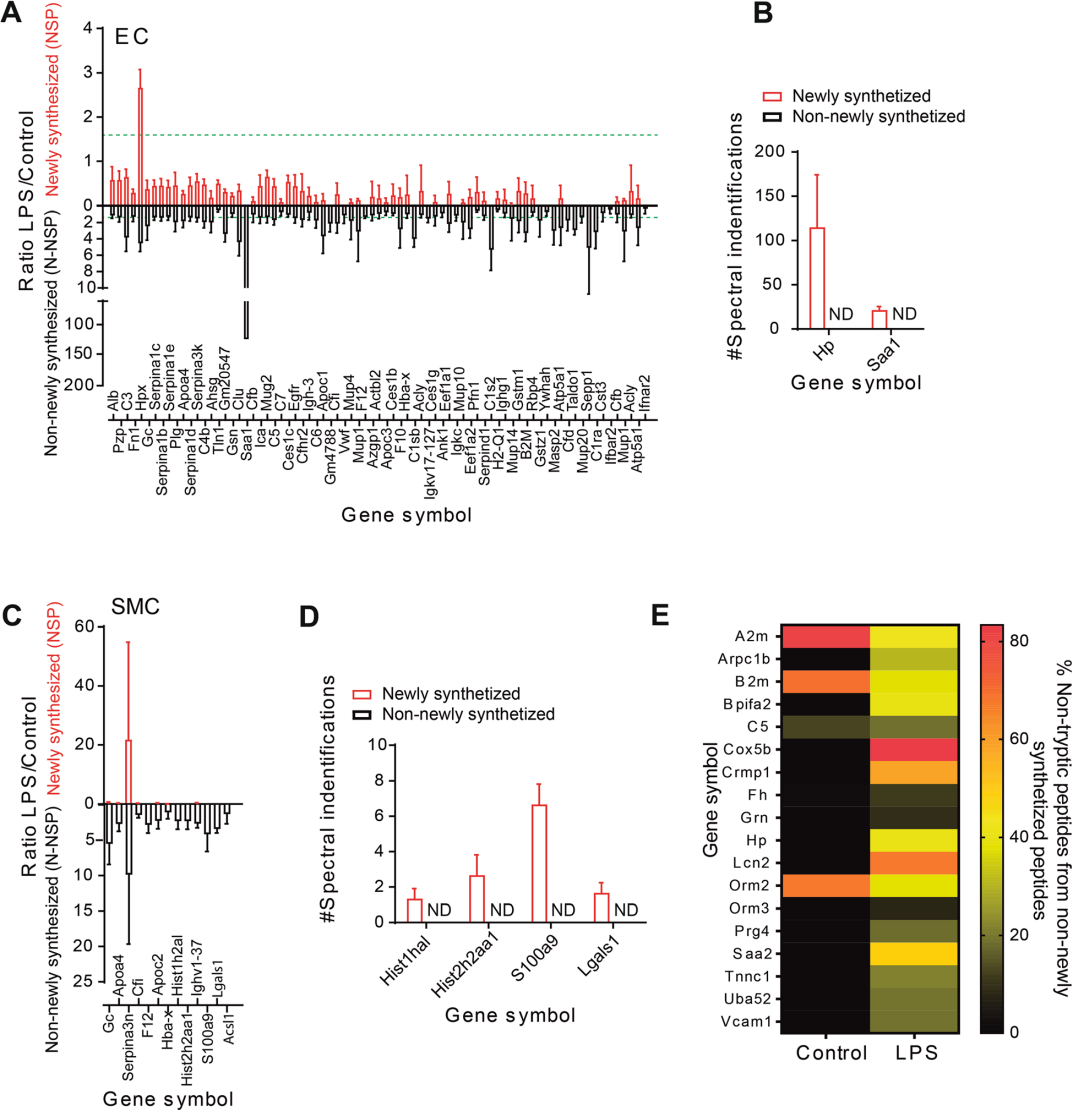
Proteome-wide modulation analysis in severe inflammatory response. **a** Representation of the LPS/Control ratio for newly synthesized peptides (red columns) and non-newly synthesized peptides (black columns) in the endothelial cells (EC). **b** Proteins with newly synthesized peptides only detected after LPS challenge in EC. **c** Representation of the LPS/Control ratio for newly synthesized peptides (red columns) and non-newly synthesized peptides (black columns) in the smooth muscle cells (SMC). **d** Proteins with newly synthesized peptides only detected after LPS challenge in SMC. Ratios were calculated based on the sum of spectral counts of all SILAM-labeled peptides for newly synthesized proteins and the sum of spectral counts of all non-SILAM-labeled peptides for non-newly synthesized proteins for every protein detected. N.D. refers to not detected. Only proteins with statistical significance assessed by Student’s *t* test are represented (*p* < 0.05). Regulation threshold has been set at 1.5 and it is represented with horizontal green dashed lines in every condition. *Y*-axis for black columns with positive values has been drawn pointing down for visual purposes. **e** Heatmap of protein turnover detected in EC. Turnover of proteins is expressed as the number of non-tryptic peptides detected in individual proteins expressed in spectral counts. Darker colors refer to lower turnover levels

The frequency of Lys(6) incorporation in newly synthesized proteins in Gram-negative sepsis was also investigated. The obtained data showed narrowing of the cumulative frequency curves of the proteomes in LPS-treated vascular beds (Fig. 1c–i). This result clearly indicates the reduced incorporation of Lys(6) into individual proteins in all of the analyzed vascular beds during Gram-negative sepsis (Fig. 1c–i). To further scrutinize this finding, the LPS/Control ratios for newly synthesized proteins (NSP_LPS/Control_) and non-newly synthesized proteins (N-NSP_LPS/Control_) were calculated for all EC and SMC proteomes. In EC, all endotoxin-modulated proteins (*p* ≤ 0.05) showed a dramatically downregulated NSP_LPS/Control_ ratio (Fig. 2a, b), except for hemopexin precursor protein (Hpx), which was highly upregulated in LPS-treated EC (Fig. 2a, b). Our data also demonstrated that SMC proteomes were less affected by the effects of endotoxin-induced sepsis on protein synthesis compared to EC proteomes, as can be observed through the reduced clustering of modulated proteins in these specific vascular beds (Fig. 2c, d). The NSP_LPS/Control_ ratio was similarly downregulated in SMC for all endotoxin-modulated proteins, as shown in Fig. 2c, except for the protein serine protease inhibitor (Serpina3n).

In a related vein, we found that endotoxemia triggered new synthesis of a specific subset of proteins in EC and SMC during Gram-negative sepsis, as shown in Fig. 2b, d. Proteins exclusively synthesized in LPS-challenged EC vascular beds included haptoglobin (Hp) and serum amyloid A1 (Saa1) (Fig. 2b), whereas S100 calcium-binding protein A9 (S100a9), galectin-1 (Lgals1), and a small cluster of histones were exclusively synthesized in challenged SMC, as shown in Fig. 2d.

The N-NSP_LPS/Control_ ratio was initially expected to be close to 1; however, we found that this ratio was strikingly modulated in a wide range of proteins in EC during Gram-negative sepsis, as shown in Fig. 2a. Further investigation of this subset of EC-specific modulated proteins indicated the increased abundance (or exclusive presence) of nontryptic peptides derived from these proteins in LPS-challenged animals, which, based on previous findings [29], clearly indicated the occurrence of active protein turnover in EC during Gram-negative sepsis (Fig. 2e).

Proteins with high turnover rates included vascular cell adhesion molecule 1 (VCAM1), the inflammation-related protein serum amyloid A (Saa2), and cytochrome c oxidase subunit 5B (Cox5b). On the other hand, the protein turnover rates of alpha-2-macroglobulin (α2M), β2 microglobulin (β2M), and orosomucoid 2 (Orm2) were found to be significantly downregulated in LPS-challenged EC vascular beds (Fig. 2e).

### Functional characterization of GC molecular dynamics

**Fig. 3:**
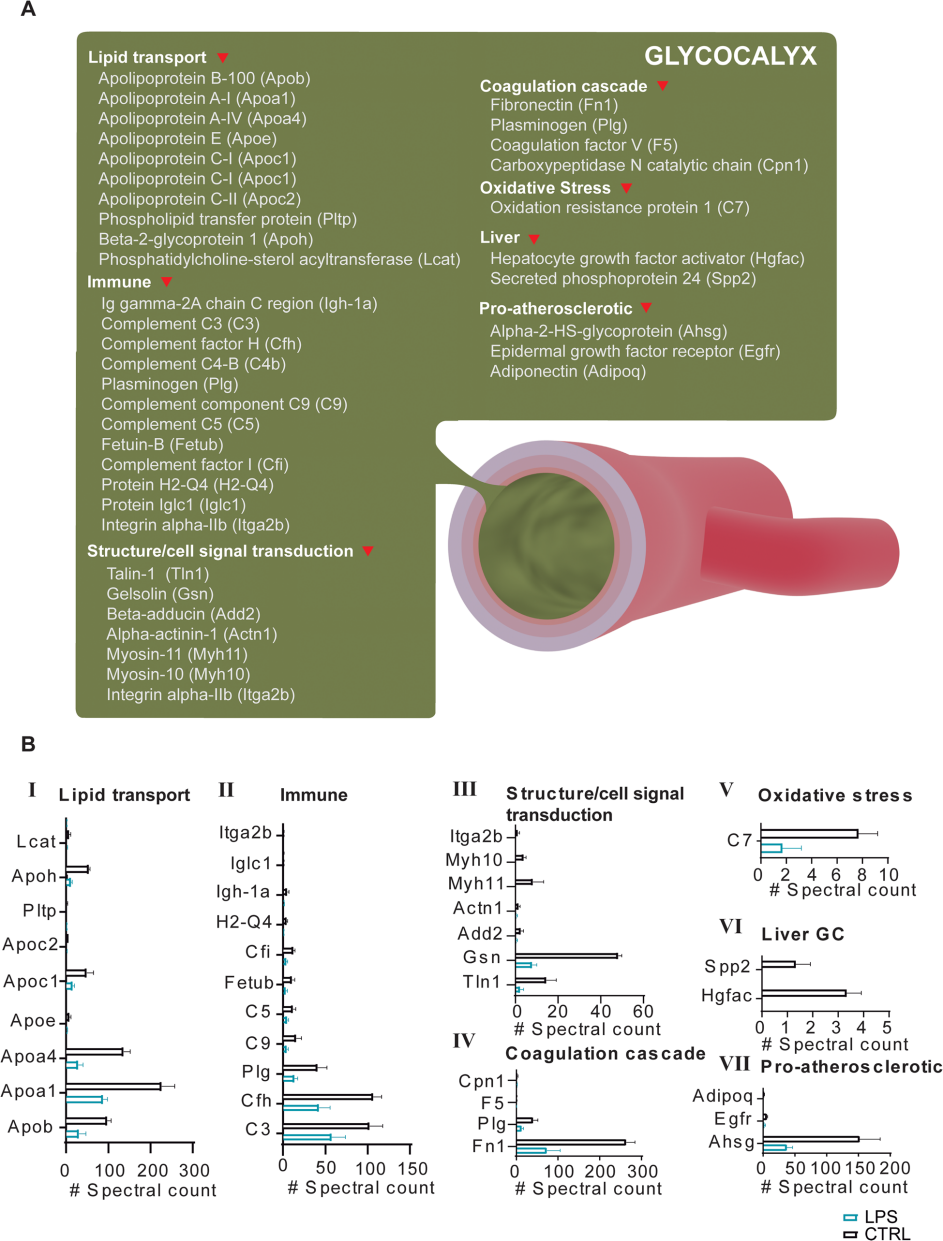
Functional analysis of molecular dynamics in severe inflammatory response for proteins identified from glycocalyx (GC). **a** Functional categorization of newly synthesized proteins from GC after a severe inflammatory response. **b** Relative quantitation of proteins included in the functional categorization. Quantitation of proteins is expressed as spectral counts considering all identified newly synthesized peptides (SILAM-labeled peptides) for every protein. Only proteins with statistical significance assessed by Student’s *t* test are represented (*p* < 0.05)

Although the GC in whole body vascular beds functions as a vasculature mantle that lacks protein synthesis ability, the GC is predictably one of the most variably affected vascular layers during Gram-negative sepsis. Thus, to further characterize any potential abnormal incorporation of newly synthesized proteins into the GC, we performed an in-depth characterization of GC Lys(6)-labeled proteomes. These analyses, as expected, revealed a reduction in proper GC molecular maintenance, which was linked to a significantly decreased abundance of important cellular mechanism-related proteins (Fig. 3a, b). These included lipid transport apolipoproteins (Fig. 3b-I), immune-related proteins (Fig. 3b-II), including fetuin-B (fetub) and integrin alpha-IIb (Itga2b), and several component proteins. Other affected GC cellular mechanism-related proteins included pro-atherosclerotic proteins, oxidative stress-related proteins, coagulation cascade proteins, and, of note, abnormally incorporated, newly synthesized structural/cell signal transduction proteins, among others (Fig. 3a, b-III to VII).

### Functional characterization of EC and SMC molecular dynamics

**Fig. 4:**
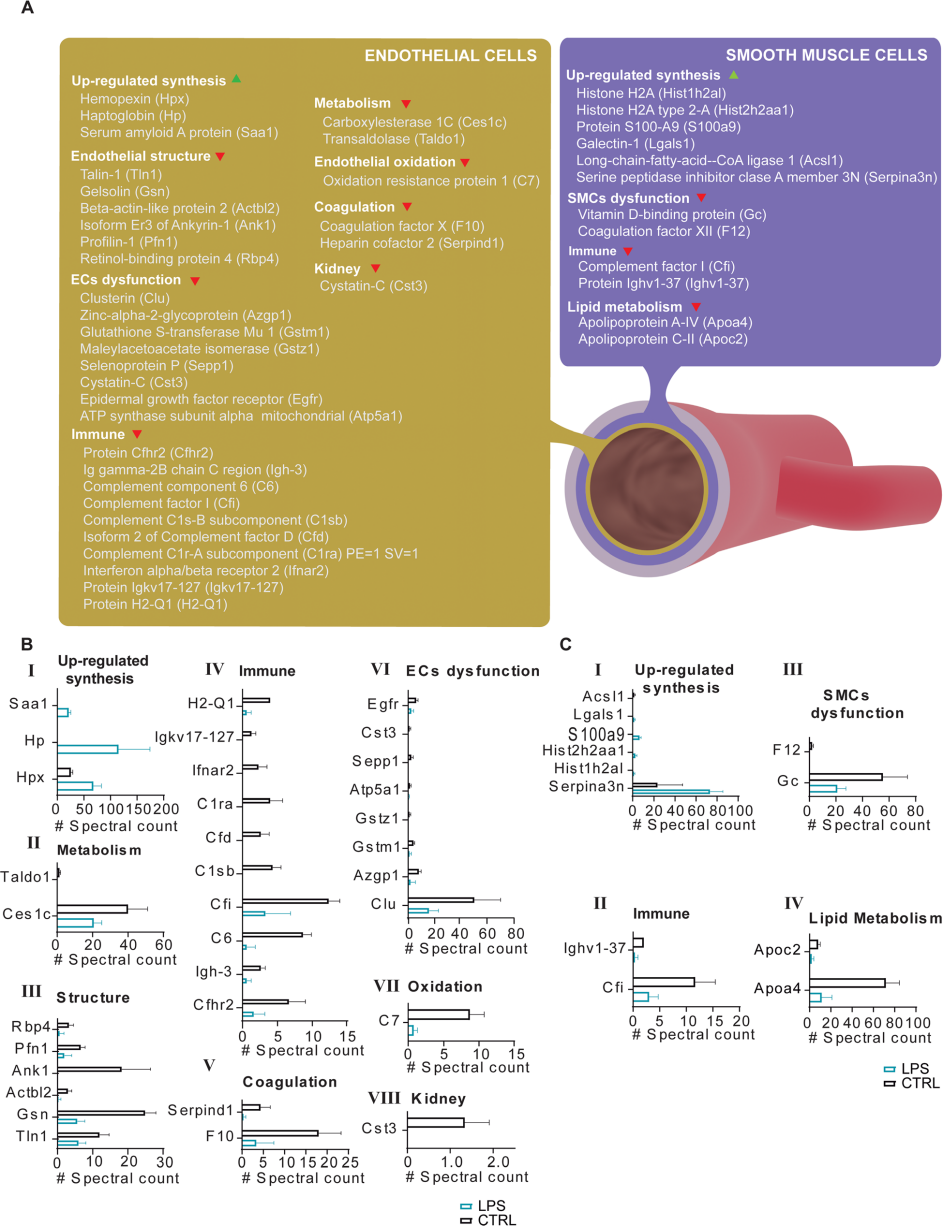
Functional analysis of molecular dynamics in severe inflammatory response for proteins identified from endothelial cells (EC) and smooth muscle cells (SMC). **a** Functional categorization of newly synthesized proteins identified by DISDIVO after a severe inflammatory response. The relative quantitation of proteins included in the functional categorization for EC and SMC are displayed in sections **b** and **c**, respectively. Quantitation of proteins is expressed as spectral counts considering all identified newly synthesized peptides (SILAM-labeled peptides) for every protein. Only proteins with statistical significance assessed by Student’s *t* test are represented (*p* < 0.05)

To investigate the most dramatically affected cellular mechanisms in EC and SMC in endotoxin-induced sepsis, we analyzed the Lys(6)-labeled EC and SMC proteomes from LPS-challenged and control mice by systems biology, as shown in Fig. 4a, b-I to VIII. The initiation of EC dysfunction was clearly induced by the downregulation of the synthesis of multiple key proteins, including clusterin (Clu), glutathione S-transferase Mu 1 (Gstm1), and selenoprotein P (Sepp1), among others (Fig. 4b-VI). Similarly, the synthesis of endothelial oxidation- and endothelial structure-related proteins was also negatively modulated (Fig. 4b-III and VII). Other cellular mechanisms significantly affected by the endotoxemic challenge included coagulation, which experienced the significant downregulation of the synthesis of coagulation factor X (F10) and heparin cofactor 2 protein (Serpind1) (Fig. 4b-V), and metabolism-related proteins through the downregulation of carboxylesterase 1C (Ces1c) and transaldose (Taldo1) synthesis (Fig. 4b-II). It is also worth mentioning the downregulation of novel protein synthesis specifically affecting the kidney-related protein cystatin-C (Cst3) in LPS-challenged EC (Fig. 4b-VIII).

To a lesser extent, multiple molecular mechanisms altered in EC during Gram-negative sepsis were also similarly affected in SMC, as shown in Fig. 4a. The cellular proteins affected by the impairment of novel protein synthesis include lipid transport-related proteins and proteins implicated in inflammatory processes (Fig. 4a). Signs of specific SMC dysfunction were also evident during Gram-negative sepsis via the downregulation of the synthesis of the proteins vitamin D-binding protein (Gc) and coagulation factor XII (F12) (Fig. 4c-I to IV).

Moreover, we observed the upregulated synthesis of hemopexin, haptoglobin, and serum amyloid proteins in the proteomes of EC in whole organism vascular beds (Fig. 4a, b-I) and of the pro-inflammatory proteins galectin-1 and S100-S9 in SMC together with several histones and serine peptidase inhibitors (Fig. 4a, c-I).

### Molecular dynamics of EC phosphoproteomes during Gram-negative sepsis

**Fig. 5:**
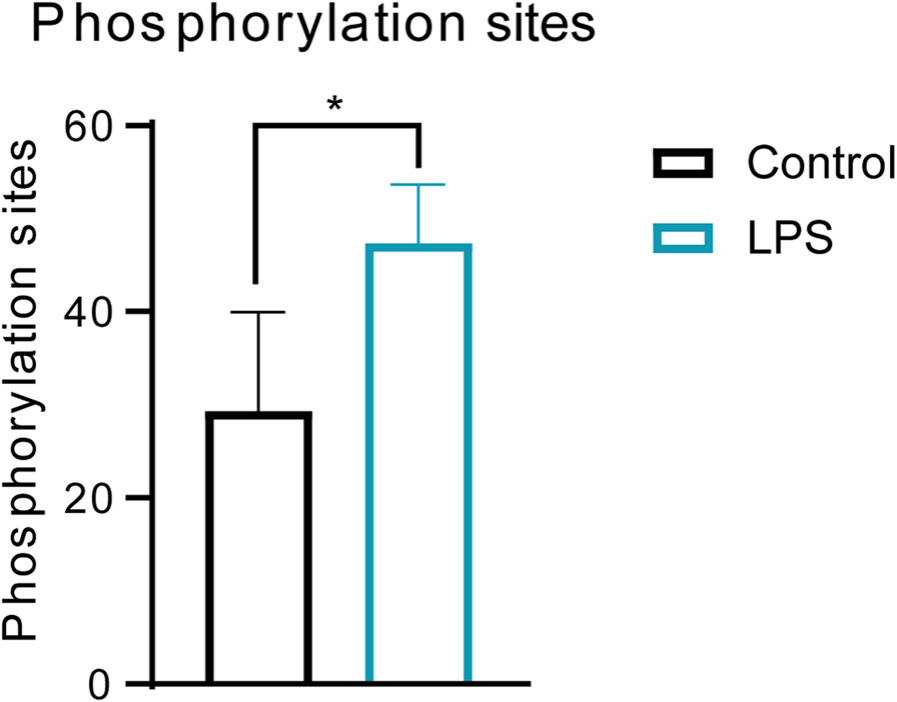
Number of total phosphorylation sites detected in Gram-negative induced sepsis in EC. The asterisk refers to significant differences observed between groups, assessed by Student’s *t* test (*p* < 0.05)

The modulation of newly synthesized proteins specifically affected by protein posttranslational modifications (PTMs) during Gram-negative sepsis was also investigated in EC and SMC in whole organism vascular beds. This part of the study revealed that significant modulation of PTMs in newly synthesized proteins was only identified in EC and in proteomes affected by PTM phosphorylation (Fig. 5). Thus, the significant upregulation of the phosphorylation of protein sites was observed in LPS-challenged animals compared to that in sham controls, as shown in Fig. 5. Furthermore, our data indicated that essential EC proteins such as VCAM1 and creatine kinase M-type contain disease-specific phosphorylation sites in Gram-negative sepsis, as detailed in Table 1.

**Table 1:** Phosphorylated proteins identified exclusively in Gram-negative sepsis in EC proteome. *Information about domains and structure for every protein was obtained from Uniprot. Numbers in brackets indicate the localization of the protein regions referred based on the amino acids’ position in the protein sequence

Gene symbol	Protein name	Modified residue	Post-translationally modified region*
Sptb	Spectrin beta chain erythrocytic	S1061, S1078, S2323	–
Cp	Ceruloplasmin	T83	Chain (20–1061), F5/8 type A 1 Domain (20–356), Plastocyanin-like 1 (20–199)
Hp	Haptoglobin	S210, S239	Polypeptide chain (19–347), Peptidase S1 domain (103–345)
Ckm	Creatine kinase M-type	T208, S224	Phosphagen kinase C-terminal domain (125–367)
Nsfl1c	Isoform 3 of NSFL1 cofactor p47	T108, S116	Before and after the nuclear localization signal motif (109–115)
Serpina1d	Alpha-1-antitrypsin 1–4	S300	Alpha-1-antitrypsin 1–4 Chain (25–413)
Vcam1	Vascular cell adhesion protein 1	Y113, S114	Extracellular domain (25–698)—next to I-set domain (C-terminal)

## Discussion

In this work, the combination of SILAM mice [25] with DISDIVO [27] allowed us for the first time to evaluate changes in the molecular dynamics of whole organism vascular beds during severe inflammation linked to Gram-negative sepsis. Our data initially showed the rapid and characteristic disruption and shedding of the pericellular apical coating of the vasculature, known as the GC. This expected finding was in line with that of previous reports [30–33]; however, in this particular case, we additionally found a global decrease in the incorporation of newly synthesized proteins into the GC, which indicates the inhibition of molecular maintenance affecting this vascular coating. Furthermore, negative modulation of phospholipid transfer protein (Pltp) coupled with downregulation of a subset of lipoproteins uncovered specific target molecules contributing to the imbalance in the transfer of essential lipid molecules to this apical vascular layer. Negative regulation of multiple pro-atherosclerotic proteins, complement factors, and plasmatic enzymes was also identified as affecting the GC in our study, which is consistent with previous reports aimed at identifying the molecular basis of GC disruption [34]. The novel data obtained here regarding the molecular dynamics and composition of the GC in Gram-negative sepsis directly advances our knowledge of the role(s) of specific proteins in vascular permeability [35]. The clinical significance of conducting further research aimed at finding novel biological markers that could detect the shedding of the GC was recently pointed out [34]. The GC has the capacity to act as a molecular target for leukocytes and inflammatory mediators, and due to its systemic nature, this mantle is one of the most fragile vascular settings that is highly targeted by endotoxemia, as observed here and in previous reports [27, 31, 36]. The novel-specific proteins linked to shedding and impaired molecular maintenance of the GC, as identified here, require further research to be established as diagnostic/prognostic markers of vascular permeability and endothelial dysfunction in Gram-negative sepsis and in other diseases that involve severe inflammation of the vasculature.

Significant impairment of protein synthesis in the EC and SMC proteomes was also found in this study. Impairment of the molecular dynamics of hindlimb muscle cells was previously reported due to Gram-negative sepsis [37] and has been very recently identified in platelet cells [23]. However, to the best of our knowledge, this has not been previously investigated in whole organism EC and SMC proteomes. Of note, Middleton et al. found highly similar sepsis-induced impairment of protein synthesis in platelet cells between murine and human samples [23]. Similarly, Vary et al. found that sepsis-induced impairment of protein synthesis in platelet cells was associated with the effect of peptide chain initiation and an increased number of free ribosomal subunits in muscle tissue [37]. Our systems biology approach, as expected, revealed the different mechanisms affecting the molecular dynamics in EC in Gram-negative sepsis. We observed that global protein turnover was significantly upregulated together with the drastic downregulation of protein synthesis affecting EC. These findings in whole organism vascular beds were in line with the catabolic phenotype observed in skeletal muscles from sepsis patients and other critically ill subjects [38]. It has been reported that muscle cells activate the inhibition of protein synthesis together with an increase in protein turnover, which encompasses a progressive and rapid decrease in muscle mass resulting in severe weakness [39]. Nonetheless, although that muscle catabolic phenotype has been described as a consequence of abnormal insulin metabolism and cytokine mediation [40], none of these metabolic processes were yet defined as modulated in the vascular beds of Gram-negative sepsis; with the exception of selenoprotein P, a protein closely related to insulin metabolism [41], which was significantly downregulated in EC, a fact that has been previously associated with the severity of sepsis and other critical illnesses [42, 43].

**Fig. 6:**
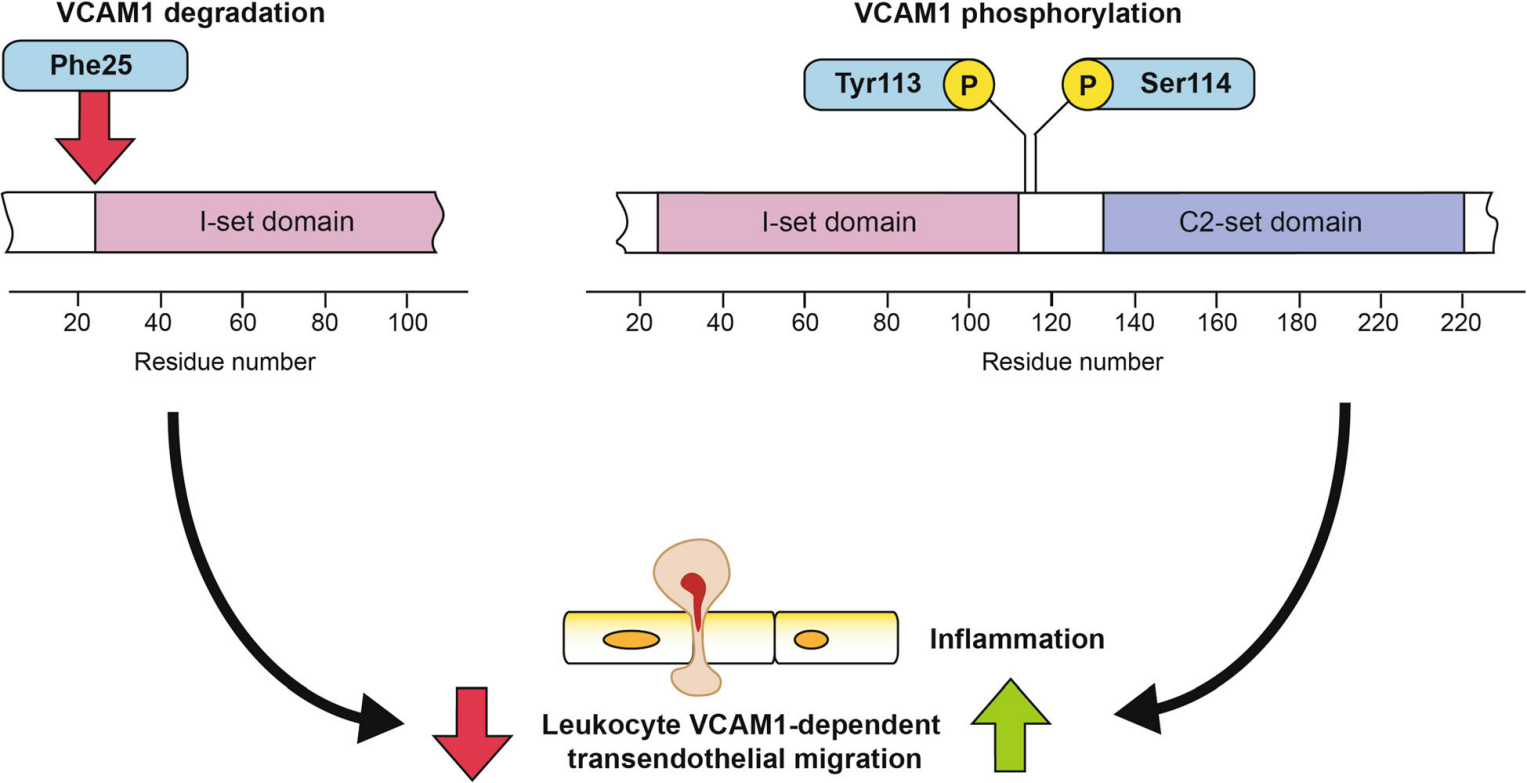
Illustrative diagram showing the identified inflammatory molecular mechanisms of VCAM1 in EC during Gram-negative induced sepsis. VCAM1 in EC contains disease-specific phosphorylations at Tyr113 and Ser114 in pro-inflammatory processes of Gram-negative-induced sepsis. Additionally, the protein is actively degraded at Phe25, which might indicate resilience of EC during systemic pro-inflammatory processes affecting the vasculature

Careful dissection of the identified catabolic phenotype affecting EC uncovered the involvement of key endothelial-specific proteins such as VCAM1. Activated VCAM1 is directly involved in the transendothelial migration of leukocytes [44], and it has been shown that its ubiquitination alters this pro-inflammatory mechanism. Here, we found ongoing direct degradation of VCAM1 at the same time that the protein is potentially activated via disease-specific phosphorylation of the Tyr113 and Ser114 residues, which are located in the region contiguous to the I-7 domain, as shown in Fig. 6. Li et al. [45] recently demonstrated that the degradation of VCAM1 in the pulmonary endothelium is directly linked to improved survival in Gram-negative sepsis. Here, we demonstrate for the first time that this is a systemic process that takes place in whole organism capillary beds in Gram-negative sepsis. This molecular mechanism, as shown in Fig. 6, serves as one of the key pathological mechanisms that sustain/cause severe inflammation in sepsis and potentially in other pathological inflammatory processes affecting the vasculature, a fact that requires follow-up research with potential highly significant clinical implications. In addition to VCAM1, other proteins containing disease-specific phosphorylated sites were also identified in this study. These proteins included spectrin beta chain erythrocytic protein (Sptb) and creatine kinase M-type, among others. Phosphorylation affecting the latter protein, creatine kinase M-type, has been linked to dynamic activation of the protein in the endothelium [46].

Detailed analysis of the molecular dynamics that take place during Gram-negative sepsis also confirmed the upregulation of the synthesis of serum amyloid A protein (Saa1) as one of the main inflammatory proteins activated in EC. This severe phase protein is generally elevated in blood during inflammation [47]. Multiple prothrombotic proteins were also upregulated in vascular beds together with the downregulation of various coagulation cascade-related proteins, including the coagulation factors F10 and F12. Activation of inflammatory and microthrombotic mediators, as encountered in EC in this study, is closely linked to sepsis endotheliopathy and seems to lead to the development of a series of fatal conditions, including thrombocytopenia, microangiopathic hemolytic anemia, and multiorgan dysfunction syndrome [39]. In addition, our protein dynamics investigation also indicated the activation of multiple protective innate mechanisms, particularly in EC. These innate protective mechanisms included the upregulation of the antithrombotic heme-binding plasma glycoprotein hemopexin (Hpx) [48] and the upregulation of the anti-inflammatory acute phase protein haptoglobin (Hp) [49] coupled to the downregulation of the anti-thrombotic and immune-related protein heparin cofactor 2 (Serpind1). The protective capacity of Serpind1 was associated with the presence of proteolytic fragments of the protein with antimicrobial and anti-coagulant capacities [50]; such fragments were also encountered in this study and linked to the downregulation of the protein, a fact that further confirms the protective and compensatory nature of the finding in whole organism capillary beds during Gram-negative sepsis. Furthermore, we consider that confirmation of the protective nature of the proteolytic Serpind1 fragments identified here paves the way for future systemic therapeutic interventions for sepsis using specific Serpind1 fragments, which requires specific investigation based on the findings reported here.

Impairment in the molecular dynamics of whole organism vascular beds also affected SMC, which is in line with the described effects on EC, although the effect on SMC was observed to a lesser extent. Downregulation of the synthesis of key proteins, such as vitamin D binding protein and coagulation factor XII, was encountered in SMC proteomes. This fact, however, further confirms that Gram-negative sepsis has major effects on whole organism capillary beds, which explains the exacerbated effect on the molecular dynamics of EC compared with that of SMC.

## Conclusions

Gram-negative sepsis has been widely used to model sepsis, and it is accepted that LPS exposure is an important part of this complex illness. Thus, our generated data expand on the previously limited knowledge about how protein synthesis and degradation become altered in the vasculature due to a systemic inflammatory response in sepsis. Globally, our novel generated data indicate that proteome molecular dynamics become altered in EC with a major impact on whole organism capillary beds. Similarly, EC fail to maintain the proper molecular integrity of the GC by not providing that apical layer with the required newly synthesized structural proteins. Furthermore, abnormal protein turnover during Gram-negative sepsis affects essential EC proteins, such as VCAM1, and is coupled to the global downregulation of protein synthesis and the generation of disease-specific phosphorylation sites. Finally, SMC alter their molecular dynamics in line with EC, although to a lesser extent.

The findings reported here, thus, uncover for the first time specific molecules that become altered in the protein synthesis machinery of the GC and EC, which indicates that the increase in VCAM1 that is typically associated with endothelial dysfunction in several diseases, including sepsis, may be due to altered degradation of the protein and the accumulation in the endothelium of dysfunctional VCAM1. Similarly, novel Gram-negative sepsis-specific phosphorylation sites have been uncovered for the first time. These findings can serve as a foundation for future therapeutic strategies aimed at maintaining the structural and functional integrity of the vasculature in sepsis, as they can provide novel insights into the previously unknown molecular mechanisms that become altered in the vasculature due to the systemic inflammatory response, which is a pathological mechanism common to several human diseases.

## Methods

### Reagents

All reagents were purchased from Sigma-Aldrich (St. Louis, MO) unless otherwise specified. Sequencing-grade modified trypsin was purchased from Promega (Madison, WI).

### Animals

Ten-week-old male C57BL/6NT mice were housed in cages on a 12-h dark/light cycle at stable temperature (21 °C) with water provided ad libitum and fed with standard commercial chow for a minimum of 2 weeks (adaptation period) before starting the experimental part. All experimental procedures were approved by the Nanyang Technological University Institutional Animal Care and Use Committee (IACUC) and were performed humanely and in strict accordance with the International Guiding Principles for Animal Research. The 3Rs principle in animal experimentation [51] was in all cases observed.

### Sepsis-induced inflammation model under stable isotope-labeled diet

Mice were maintained in fasting conditions for 16 h before being exposed to a stable isotope-labeled diet (Lys(6)-SILAM-Mouse diet, pellet ø 10 mm, Silantes GmbH, München, Germany; *n* = 6). As the effect potentially caused by an acute treatment with LPS on the protein dynamics of the endothelium was expected to be strong, as it was later identified, we kept the number of animals used in the experiments to the minimum that allowed to identify outliers and to obtain statistical significance, as previously recommended [52]. Mice were kept in SILAM diet for 24 h before treatment and were then divided into 2 groups (control and LPS treated). LPS treated mice were injected with a total of 20 mg/kg of lipopolysaccharide [27, 53] derived from *Escherichia coli* O55:B5 freshly prepared in sterile PBS (vehicle solution). LPS was administered in two equal doses of 10 mg/kg in a 24 h interval to ensure proper intake of SILAM stable isotope-labeled chow. LPS mice were maintained in SILAM diet during all experimental procedures. Similarly, control mice were injected with vehicle solution in a 24-h interval and maintained in SILAM diet during the whole experimental procedures.

### DISDIVO obtention of whole organism vascular beds

Systemic isolation of vascular beds was carried out by differential systemic decellularization in vivo (DISDIVO) as previously described [27]. Briefly, mice were anesthetized by intraperitoneal injection with ketamine-xylazine (90:10 mg/kg) and deep anesthesia was maintained over the whole experimental procedure by inhalation of isoflurane (IsoFlo; Veterinaria Esteve, Bologna, Italy). For the DISDIVO procedure, an exsanguination by transcardial whole-body perfusion with open right auricle was performed while 1× PBS was simultaneously introduced at a flow rate of 1.5 mL/min through the left ventricle. PBS perfusion was maintained for 1.5 min after complete removal of blood when the collection of PBS fraction from the open right auricle was initiated and maintained over 3 to 4 min to collect the GC-containing outflow. EC decellularization was subsequently performed perfusing with 0.5% sodium deoxycholate (SDC) prepared in 100 mmol/L ammonium acetate buffer through the entire circulatory system. EC decellularization was maintained over 3–4 min collecting the EC lysed tissue-containing outflow from the open right auricle, and subsequently, concentration of SDC was increased to 10% to decellularize SMC vascular beds. Perfusion with 10% SDC prepared in 100 mM ammonium acetate buffer was maintained over 3–4 min. All collected outflows were stored at − 80 °C until analysis.

### In-solution tryptic digestion of vascular beds proteomes

Vascular beds proteomes were digested by in-solution digestion as previously described [27, 54]. Briefly, DISDIVO outflows were adjusted to 1% SDC using a 10% SDC stock solution prepared in 100 mM ammonium acetate for GC and EC fractions or by dilution with 100 mM ammonium acetate for SMC fractions. Vascular beds proteins were subsequently reduced using 10 mmol/L dithiothreitol (DTT) for 30 min at 60 °C and alkylated using 20 mmol/L iodoacetamide for 45 min at room temperature protected from the light. Samples were then 2-fold diluted with 10 mmol/L DTT prepared in 100 mmol/L ammonium acetate and incubated for 30 min at 37 °C. Tryptic digestion was performed at 30 °C overnight using sequencing-grade-modified trypsin at 1:50 (w/w) enzyme-to-protein ratio. Enzymatic digestion was quenched by addition of a final concentration of 0.5% formic acid (FA) and SDC salts were precipitated by acidification. Peptide recovery from precipitated SDC was performed as follows: SDC was pelleted by centrifugation at 12,000*g* for 10 min at 4 °C. The supernatant containing peptides was then separated and pelleted SDC was redissolved in 0.5% ammonium hydroxide before reprecipitation with 0.5% FA. Peptide recovery was performed per duplicate and supernatant combined. Peptides were desalted using a C18 Sep-pack cartridge (Waters, Milford, MA). Eluates were finally dried in a vacuum concentrator (Eppendorf, Hamburg, Germany).

### High-pressure liquid chromatography fractionation of vascular beds proteomes

Vascular beds desalted peptides were fractionated by high-pressure liquid chromatography as previously described [55]. Briefly, dried samples were reconstituted in 200 μL of 10 mmol/L ammonium hydroxide in water (mobile phase A) and separated using a XBridge BEH130 C18, 3.5 μm, 4.6 × 250 mm column (Waters, Elstree, UK) on a Shimadzu Prominence UFLC system (Dionex, Sunnyvale, CA) monitoring UV of peptide intensities at 280 nm. Peptide separation was performed over a 72-min gradient at 1 mL/min as follows: 0% mobile phase B (10 mmol/L ammonium hydroxide in acetonitrile) for 5 min, 0% to 20% for 30 min, 20% to 33% for 15 min, 33% to 60% for 10 min, and 60% to 100% for 5 min, followed by 7 min at 0% mobile phase B. Fractions were collected every minute and combined by concatenation. Combined fractions were completely dried in the vacuum concentrator.

### Liquid chromatography tandem-mass spectrometry analysis of vascular beds proteomes

Dried fractionated peptides were reconstituted in 3% acetonitrile (ACN), 0.1% FA (mobile phase A), and analyzed by liquid chromatography tandem-mass spectrometry (LC-MS/MS) using a Dionex UltiMate 3000 UHPLC system coupled with an Orbitrap Elite mass spectrometer (Thermo Fisher, Inc., Bremen, Germany) [56, 57]. The sample was sprayed using a Thermo Fisher Easy-Spray source working at 1.5 kV and separated using a reverse-phase Acclaim PepMap RSL column (75 μm ID × 15 cm, 2-μm particle size; Thermo Scientific, Inc.) maintained at 35 °C and working at 300 nL/min. Peptides were separated over a 60-min gradient as follows: 3% mobile phase B (90% acetonitrile, 0.1% FA) for 1 min, 3% to 35% for 47 min, 35% to 50% for 4 min, 80% for 6 s, 80% (isocratic) for 78 s, 80% to 3% for 6 s, and then maintained at 3% (isocratic) for 6.5 min. Data adquisition using Xcalibur 2.2 SP1.48 software (Thermo Fisher Inc., Bremen, Germany) was performed in positive mode alternating between full Fourier transform mass spectrometry (FT-MS; 350–2000 m/z, resolution 60,000, 1μscan per spectrum) and FT-MS/MS (150–2000 m/z, resolution 30,000, 1μscan per spectrum). The 10-most intense ions with charge > + 2 were isolated within a 2-Da window and fragmented by high-energy collisional dissociation mode using 32% normalized collision energy with a threshold of 500 counts. Automatic gain control was set to 1 × 10^6^ for FT-MS and FT-MS/MS.

### Bioinformatics and data analysis

Database search of raw proteomics data obtained from the LC-MS/MS analysis was analyzed using PEAKS Studio version 7.526 (Bioinformatics Solutions, Waterloo, Canada) as previously described [58, 59] with minor modifications. The database search was performed using an ion tolerance of 10 ppm and a fragment ion tolerance of 0.05 Da. The false-discovery rate used was 1% [60]. Carbamidomethylation at Cys was set as fixed modification and SILAC K6 (+ 6.0201 Da) at Lys was set as variable modification. The UniProt mouse database (58,761 entries; downloaded on February 18, 2016) was used for searching. Only proteins consistently identified in at least 2 animals were considered. Identification of protein post-translational modifications (PTMs) was carried out using PEAKS PTM algorithm and only PTM manually validated and with an Ascore of 1000 were considered in this study. Obtained raw data were analyzed in Microsoft Excel with the help of in-house created macros. GraphPad Prism 8 (GraphPad Software, Palo Alto, CA) was used for statistical analyses of results and creation of data plots. Statistical significance was established by ANOVA followed by Bonferroni post hoc multiple comparisons at *P* < 0.05, unless otherwise specified. Data are reported as mean ± SD, unless stated otherwise. Illustrations were created using the open-source software Blender version 2.8 [61] and Adobe Illustrator CS5.

## Supplementary Information

**Additional file 1.** List of peptides identified in DISDIVO-isolated vascular beds (GC, ECs and SMCs) from LPS-treated mice. Replicate for every vascular bed analyzed are included in different Tabs (1 to 3).

**Additional file 2.** List of peptides identified in DISDIVO-isolated vascular beds (GC, ECs and SMCs) from Control mice. Replicate for every vascular bed analyzed are included in different Tabs (1 to 3).

## Data Availability

All data generated in this study have been made publicly available as indicated below: Authors: Xavier Gallart-Palau, Aida Serra, Siu Kwan Sze Publisher: ProteomeXchange via repository PRIDE Title: Characterization of proteome dynamics in individual vascular layers at the early stage of acute sepsis Accession: PXD018274 Project Webpage: http://www.ebi.ac.uk/pride/archive/projects/PXD018274 FTP Download: ftp://ftp.pride.ebi.ac.uk/pride/data/archive/2020/10/PXD018274

## Abbreviations

ACN: Acetonitrile
CVS: Cardiovascular system
DISDIVO: Differential systemic decellularization in vivo
DTT: Dithiothreitol
EC: Endothelial cells
FA: Formic acid
GC: Glycocalyx
LC-MS/MS: Liquid chromatography tandem-mass spectrometry
LPS: Lipopolysaccharide
NSP: Newly synthesized proteins
N-NSP: Non-newly synthesized proteins
PTM: Post-translational modifications
SDC: Sodium deoxycholate
SILAM: Stable isotope labeling of mammals
SMC: Smooth muscle cells
